# Molecular Imaging in Ischemic Heart Disease

**DOI:** 10.1007/s12410-019-9500-x

**Published:** 2019-06-11

**Authors:** Begoña Lavin Plaza, Iakovos Theodoulou, Imran Rashid, Reza Hajhosseiny, Alkystis Phinikaridou, Rene M. Botnar

**Affiliations:** 1 School of Biomedical Engineering and Imaging Sciences, King’s College London, 3rd Floor, Lambeth wing, St Thomas Hospital, London, SE1 7EH UK; 2 Faculty of Life Sciences and Medicine, King’s College London, London, UK; 3 Escuela de Ingeniería, Pontificia Universidad Católica de Chile, Santiago, Chile

**Keywords:** Cardiovascular imaging, Inflammation, Ischemic heart disease, Vascular remodeling, Myocardial infarction

## Abstract

**Purpose of Review:**

The purpose of this paper is to review current and new modalities to image key biological processes in ischemic heart disease and after myocardial infarction non-invasively.

**Recent Findings:**

New imaging targets have been developed to detect and quantify myocardial damage after ischemia. Although positron emission tomography (PET) has been leading the development of new probes in the past, continuous improvements of magnetic resonance imaging (MRI) together with the development of new novel MRI contrast agents opens new research avenues including the combination of both PET and MRI to obtain anatomic, functional, and molecular information simultaneously, which is not possible from a single imaging session.

**Summary:**

This review summarizes the state of art of non-invasive molecular imaging of the myocardium during ischemia and after myocardial infarction using PET and MRI. We also describe the different contrast agents that have been developed to image the different phases of cardiac healing and the biological processes associated with each of those phases. Importantly, here we focus on imaging of inflammation as it is the key biological process that orchestrates clearance of dead cells, tissue remodeling, cardiac repair, and future outcome. We also focus on clinical translation of some of the novel contrast agents that have been tested in patients and discuss the need for larger, multi-center patient studies to fully validate the applicability of new imaging probes.

## Introduction

Cardiovascular diseases remain the leading cause of morbidity and mortality in western societies with coronary artery disease and associated myocardial infarction (MI) being the most common type of disease in the circulatory system [1]. Myocardial infarction occurs when the coronary arteries are obstructed, limiting the transport of nutrients and oxygen to the cardiomyocytes that form part of the ventricular wall. After the ischemic event, a plethora of molecular and cellular pathways are activated to compensate and resolve the injury to the heart. A proportion of the cardiomyocytes die in response to the coronary obstruction, creating an environment that will stimulate the infiltration of inflammatory phagocytes such as neutrophils immediately after the event and is followed by the influx of inflammatory and reparative monocytes [2]. Inflammation plays a crucial role in cardiac healing, first by removing dead cells and secondly by activating cells that will contribute to the healing response which includes the production of extracellular matrix proteins that will become an integral part of the scar tissue that will replace the dead cardiomyocytes [3]. However, if the inflammatory response is unbalanced (too strong or too weak), the effect on cardiac healing can be deleterious, resulting in ventricular dilation, hypertrophy or thinning of the myocardium among others, a phenomenon known as adverse ventricular remodeling. Patients with adverse ventricular remodeling have a higher chance of developing progressive heart failure and associated poor prognosis [4, 5]. Therefore, the identification of patients undergoing adverse ventricular remodeling is crucial in influencing their therapeutic intervention and ultimately improving their prognosis.

Current clinical imaging techniques are mainly focused on assessing the anatomy and function of the heart. However, the development of new molecular-targeted probes can increase significantly the information obtained from each imaging session, by better visualization and understanding of the molecular pathways involved in myocardial healing and, thus, help clinicians to provide more personalized treatments to their patients. In this review, we focus on the two primary imaging modalities used for cardiac molecular imaging, positron emission tomography (PET) and magnetic resonance imaging (MRI), and we discuss new molecular-targeted probes used to detect different biological processes after myocardial infarction with special focus on inflammation, as it is considered the key biological process in cardiac healing.

### Clinical Need for Non-invasive Imaging of Post-MI Remodeling

Optimal myocardial remodeling after ischemia relies on a suitable degree of inflammation and its timely resolution. Inadequate recruitment of granulocytes and monocytes into the infarcted area triggers impaired healing frequently promoting adverse cardiac remodeling, impaired scar stability, and long-term risk of heart failure [4, 6•]. Blood biomarker tests including serum troponin levels, together with clinical symptoms of chest pain, and ST segment changes or T-wave inversion on the electrocardiogram (ECG) constitute the basis for identifying patients suffering from myocardial ischemia [7]. Although increased levels of inflammatory blood biomarkers, such as interleukin (IL)-6, tumor necrosis factor (TNF)-α, and C reactive protein (CRP), may predict poor prognosis, they do not provide information on the local inflammatory response triggered in the ischemic myocardial segment. Therefore, the ability to directly visualize and assess the healing process and cardiac remodeling may produce useful clinical information to help individualize patient treatment. In this review, we will focus on clinical and experimental non-invasive imaging approaches available for assessing cardiac remodeling after ischemia, with a specific focus on the inflammatory phase and macrophage imaging.

### Myocardial Remodeling After Ischemia

Coronary artery disease is the consequence of progressive and gradual narrowing of the coronary arteries by the build-up of fatty material within the vessel walls or by the obstruction of the coronary lumen by clot formation as a consequence of acute plaque rupture. A complete obstruction can severely limit the availability of oxygen and nutrients inducing rapid cardiomyocyte damage and death [8–10]. Within the culprit coronary artery myocardial territory, the subendocardial layer is damaged first, due to effects of cardiac contraction, vascular pressure-dependent compliance, and potential transmural differences in vessel anatomy [11]. If the obstruction persists, the injury will progress along a wave front starting from the subendocardial layer towards the subepicardial layer [12–14]. Therefore, the time lapse between acute coronary obstruction and revascularization directly determines the extent of myocardium injury and significantly affects long-term cardiac function and prognosis. However, the restoration of blood flow and subsequent reperfusion paradoxically induces injury in the myocardium [15–18]. Several approaches to minimize myocardial injury inflicted by reperfusion have been tested throughout processes known as pre- and post-conditioning [17, 19].

**Fig. 1:**
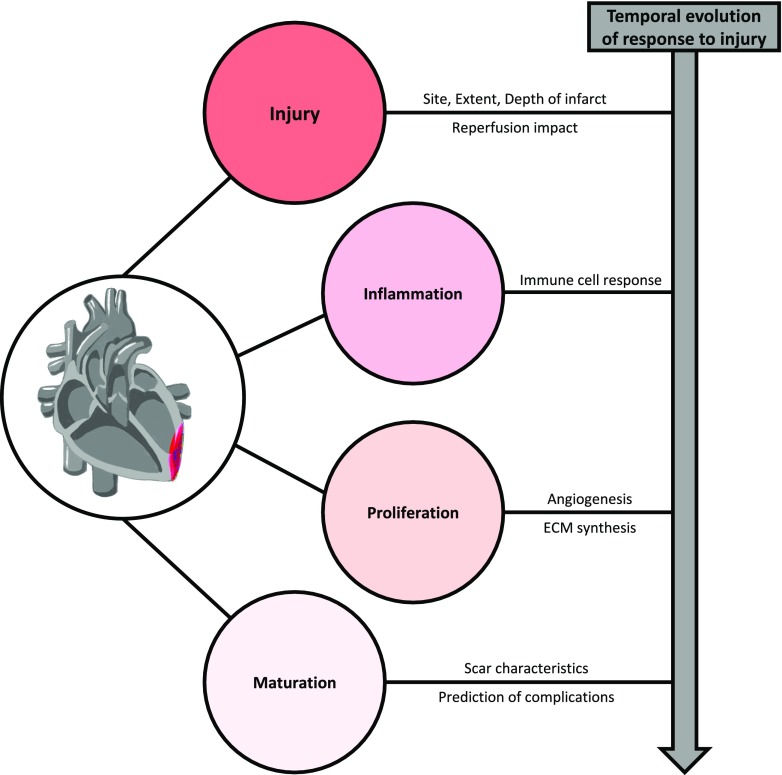
Summary of the healing phases and biological alterations that occur in the myocardium following an ischemic event

The healing of the myocardium after ischemia consists of an early inflammatory phase followed by a proliferative phase and a maturation phase [2, 20] (Fig. 1). This process involves a complex cascade of molecular, cellular, and physiological responses that affects the structure of the heart. During the early phase after the insult, inflammatory signals recruit neutrophils to the infarcted area within the first 24 h. Neutrophils and other granulocytes secrete several proinflammatory cytokines such as, IL-1β, IL-12, interferon (IFN)-γ, and TNF-α [20, 21], while damaged and dying cardiomyocytes secrete factors such as monocyte chemoattractant protein (MCP)-1 [2, 22]. All of these cytokines and chemokines potentiate the inflammatory response and trigger the recruitment of “inflammatory/classic” Ly6C^high^ monocytes that will differentiate into M1-like tissue resident macrophages, being the predominant inflammatory cell type found in the injured myocardium 3 to 5 days after the insult. M1-like macrophages secrete a broad range of inflammatory cytokines and have high protease activity being responsible for the clearance of dead cardiomyocytes and their debris and degrade the extracellular matrix, which can weaken the myocardial wall and increase the susceptibility of rupture and sudden death [2]. Around day 7, “reparative” Ly6C^low^ monocytes are recruited to the infarcted myocardium, differentiating into M2-like reparative tissue resident macrophages. At this point the healing process enters the anti-inflammatory/proliferation stage were both M2-like macrophages and endothelial cells release anti-inflammatory markers such as IL-10, vascular endothelial growth factor (VEGF), and tumor growth factor (TGF)-β. This response elicits angiogenesis that supplies blood to the infarcted area, extracellular matrix synthesis, and myofibroblast proliferation and reorganization [2, 23]. Finally, during the maturation phase, a collagen-rich scar is formed, which adequate organization is crucial for preventing heart dilation and rupture. However, the scar formed in the infarcted area has poor conductivity properties, potentially compromising the electrical activity and therefore function of the heart [24–26].

As mentioned above, the extent and duration of the inflammatory response post-MI may have profound effects on the cardiac remodeling and clinical outcome. High levels of specific inflammatory markers, such as IL-6 and TNF-α, in blood have been correlated with long-term heart failure and poor prognosis [27]. High levels of white blood cells within 24 h after ischemia have shown to be a strong predictor of 30-day mortality and recurrent clinical events [28]. The CANTOS study has demonstrated the importance of the inflammatory marker IL-6 in patients with history of myocardial infarction showing the potential beneficial effects of the modulation of its signaling pathways, thereby reducing cardiovascular event rates independently of lipid lowering [29, 30•]. Similarly, high levels of other inflammatory markers such as TNF-α and IL-10 have been associated with recurrent events and poor prognosis [31, 32]. It is also important to note that a weak inflammatory response after MI can be deleterious, as the inflammatory cells might not be able to clear up the injured area from damaged and dead cells and not be able to produce strong enough signals to activate other cells involved in the healing response and scar formation [33]. Therefore, non-invasive imaging of cardiac remodeling and more specifically inflammation after myocardial infarction can be a useful clinical tool for patient stratification allowing more targeted and individualized therapeutic approaches.

### Molecular Imaging of Cardiac Remodeling After Myocardial Infarction

Several imaging approaches can be employed to image the structural, functional, and molecular changes during persistent ischemia and after infarction. Cardiovascular magnetic resonance imaging (CMRI) is a non-ionizing imaging modality which allows the assessment of cardiac function by using cine imaging and anatomical evaluation with bright and black blood imaging. In addition, CMRI allows detailed myocardial tissue characterization using T1 and T2 mapping by exploiting the differences in T1 and T2 relaxation time of the myocardium during health and disease [34, 35]. Following myocardial infarction, the area at risk is characterized by edema, which is representative of the percentage of myocardium affected after the insult. Edematous regions are usually visualized on T2-weighted but can also be detected on T1-weighted images as both are sensitive to changes in water concentration, without the need for contrast administration [10]. Native T1 and T2 mapping are starting to replace T1w and T2w imaging as they can quantify the amount of edema and, thereby, further improve diagnostic accuracy [36]. The detection and quantification of the extent of the area at risk and especially the area of infarction can be improved using contrast agents. Gadolinium-based contrast agents can be used to determine the damaged areas by using early gadolinium enhancement (EGE) for area at risk detection and late gadolinium enhancement (LGE) for direct infarct visualization [36]. As the contrast agent washes out slower from scar tissue compared to the healthy tissue, scar appears bright on heavily T1-weighted inversion recovery prepared LGE images. In addition, myocardial perfusion can be assessed qualitatively and quantitatively using first pass MR perfusion imaging with gadolinium-based contrast agents, as damaged and poorly perfused myocardium will show less gadolinium uptake and, therefore, reduced signal on T1-weighted images [37, 38].

**Fig. 2:**
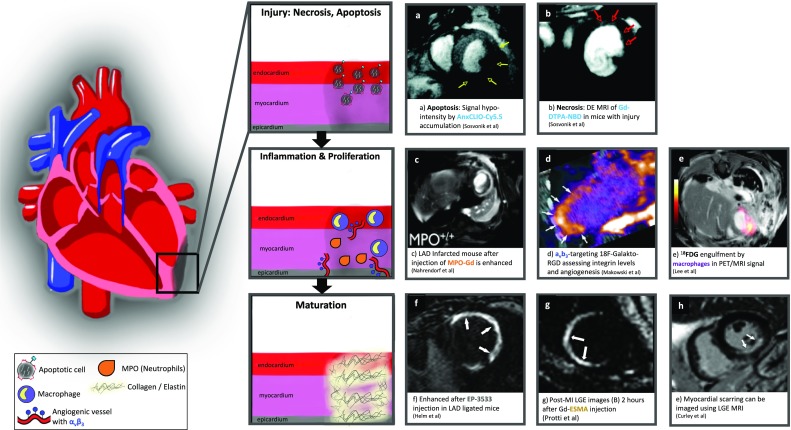
Representative examples of targeted and non-targeted contrast agents developed to evaluate biological alterations after myocardial infarction using MRI and PET

PET is a non-invasive imaging technique that uses radioactive tracers that allow detection and quantification of biological functions in healthy and more importantly, in diseased tissues. This modality uses gamma detectors that are positioned in a stationary ring around the patient which detects the two photons produced after the annihilation event between and electron and a positron produced during radiotracer decay, thereby allowing localization of the annihilation event. The acquisition of the radioactive tracer decay over several minutes allows the production of a series of dynamic images which can then be reconstructed to produce a final higher quality 3D dataset. In the following sections, we will describe some of the biological alterations that occur in the heart after ischemia and the potential of targeted and non-targeted contrast agents to image those processes with MRI and PET (Fig. 2).

### Cell Death

Immediately after myocardial infarction, a hypoxic environment is generated in the territory supplied by the obstructed coronary artery leading to cardiomyocyte damage and death. Cells generally die through a non-programmed or a programmed cell death pathway, which are known as necrosis and apoptosis, respectively. During necrosis, the cell membrane is disrupted and the cell content is released to the extracellular space, leading to the activation of the inflammatory response. However, during apoptosis, cells shrink and form apoptotic bodies exposing specific markers in their membrane that are detected and subsequently phagocytosed by macrophages.

One of the first approaches to measure myocardial apoptosis was targeting the intracellular protein myosin using indium-111 (^111^In)-labeled anti-myosin antibodies in dogs with MI [39]. Clinical validation of targeting the intracellular myosin was performed using technicium-99 (^99m^Tc) labeled anti-myosin antibodies in 30 patients that underwent percutaneous revascularization after myocardial infarction. This approach allowed imaging of the necrotic area, but not the border zones that may or may not have irreversible injury [40]. No follow-up studies have been reported since. Annexin-V is one of the markers expressed in apoptotic cells and has been used as target for non-invasive imaging of apoptosis after MI using MRI. This approach uses Annexin-V-labeled nanoparticles, known as AnxCLIO-Cy5.5, in a murine model of heart failure [41] and acute ischemia [42]. Other approaches are focused on the development of contrast agents targeting caspases, which are key proteins during the apoptotic process. However, all efforts so far have been focused on imaging apoptosis in cancer [43], with little to no work in cardiac apoptosis.

### Vascular Permeability (Endothelial Damage and Angiogenesis)

Acute ischemia is associated with damage of the endothelial cells that cover the ventricle and, therefore, an increase in vascular permeability is observed. Moreover, during the chronic maturation phase, increased levels of VEGF and basic fibroblast growth factors are released in the injured area, triggering the formation of immature or leaky neovessels contributing also to an increase in permeability in the infarcted area. Intense research efforts have been undertaken to develop contrast agents that can detect and quantify vascular permeability to better understand the cardiac healing process during the acute and chronic phase. Several different imaging targets sensitive to vascular permeability have been tested, including α_v_β_3_, VEGF, VCAM-1, and albumin.

**Fig. 3:**
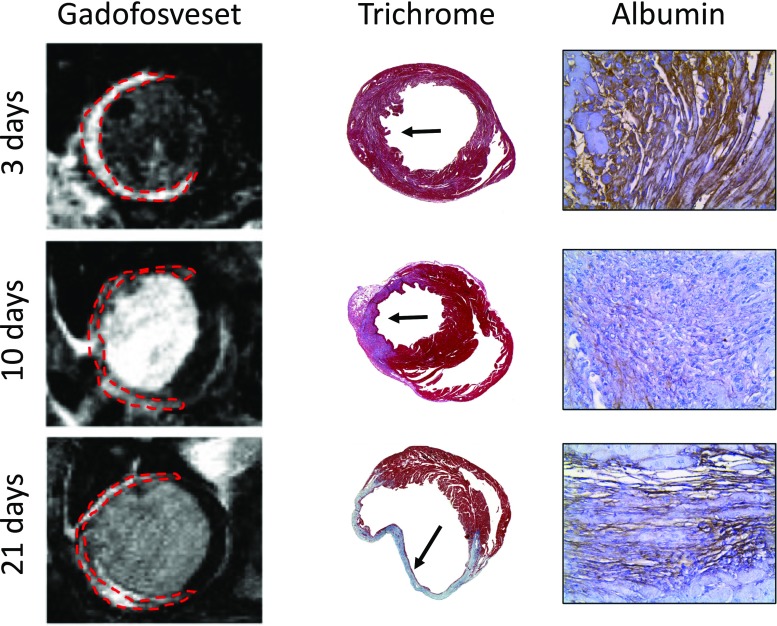
**a** Late gadolinium enhancement (LGE) images using gadofosveset showing contrast uptake in the infarcted area at different time points post-MI. **b** Trichrome images (first column) and albumin immunohistochemistry (second column) of the infarcted myocardium at different time points post-MI. Black arrow indicates the infarcted area

The α_v_β_3_ or vitronectin receptor is an integrin highly expressed in angiogenic endothelial cells that covers newly blood vessels formed during vasculogenesis or during pathological conditions such as cancer, atherosclerosis, or cardiac remodeling post-MI. However, the α_v_β_3_ integrin is not detected on mature vessels [44], making this integrin a very attractive target for the development of multiple imaging strategies and treatments for angiogenesis-related diseases. Integrins recognize a short peptide sequence known as RGD (Arg-Gly-Asp) expressed on extracellular matrix proteins and membrane surfaces [45], which is the peptide sequence mostly employed to develop imaging tracers that detect this specific integrin. The tracer ^18^F-galacto-RGD has been successfully used to image cardiac angiogenesis in animal models [46–48] and in patients after MI [49]. Gallium-based tracers have become an alternative to fluorine-based radiotracers, as the production can be performed in an onsite ^68^Ga generator as compared to the more complex chemistry and technical support needed for the fluorine-based tracers. In a rat model of MI, Laitenen et al. demonstrated that both ^68^Ga-NODAGA-RGD and ^68^Ga-TRAP(RGD)_3_ showed similar results as compared to ^18^F-galacto-RGD [9]. However, the use of these ^68^Ga-based tracers has not yet been validated in patients with MI. Similarly, approaches using ^111^In-RP748 have been validated at the preclinical level [5]. Although the research of RGD-based radiotracers and its validation in models of MI has been very intense and productive, only one RGD-based MRI contrast agent has been developed and validated in animal models of cancer and atherosclerosis [50]. MRI research has been more focused on the detection and quantification of vascular permeability using other targets. Our group has demonstrated the feasibility of imaging vascular permeability in a murine model of MI using a Gd-based albumin-binding contrast agent, known as gadofosveset, which is clinically approved as blood pool contrast agent. In this work, we demonstrated that gadofosveset allows for the detection of changes in myocardial permeability thereby allowing to differentiate between the acute and chronic phases following MI [51] (Fig. 3).

### Extracellular Matrix

The maturation or chronic phase after ischemia is characterized by the reorganization and production of new extracellular matrix proteins, the development of a fibrotic scar, typically rich in type I collagen fibers to prevent heart dilation and rupture. The presence and extent of myocardial scar carries important diagnostic and prognostic information [52–54]. LGE MRI is currently considered the standard of reference for scar detection and quantification [55]. The increase in extracellular volume (ECV) in the damaged myocardium together with the delayed wash allows gadolinium to accumulate in the infarcted region, thereby providing exquisite visualization of scar on highly T1-weighted inversion recovery LGE MR images [56]. While LGE MRI can visualize the location and extent of the scar, pre- and post-contrast T1 mapping enables the quantification of ECV non-invasively [57]. ECV has been shown to be an excellent marker for focal and diffuse fibrosis [56]. While LGE MRI and T1 mapping provide important information about scar size and extent of transmurality and the amount of fibrosis, those methods fall short in providing detailed information on the underlying biology. An alternative approach overcoming the above limitation is the use of Gd-based target-specific contrast agents such as EP-3533, which binds to collagen types I–IV and, thus, provides additional information beyond LGE MRI or T1 mapping with non-specific Gd-based agents. EP-3533 has been successfully validated in a murine model of ischemia-reperfusion and demonstrated the ability to detect collagen-rich scar tissue 6 weeks after MI [58]. Other extracellular matrix proteins that are overexpressed after MI include elastin and tropoelastin. Importantly, studies suggest that elastin formation after MI leads to improved ejection fraction and decreased risk of myocardial rupture [59] and therefore may be an attractive imaging candidate. We and other groups have demonstrated the merits of an elastin-binding contrast agent, known as ESMA, to visualize and quantify the infarcted area [60]. While the area of infarction was similar to that measured with a conventional Gd-based contrast agent, elastin imaging enabled the monitoring of changes in extracellular matrix remodeling over time (higher signal at 3 weeks post-MI) in both the infarct and remote area, which were not seen with standard LGE MRI [59, 60]. In addition, we have evaluated the interplay between elastin remodeling and inflammation after MI using simultaneous ^1^H and ^19^F imaging which may be beneficial to predicting future outcomes [61]. To enable the assessment of ECM turnover and fibrosis activity, which may be directly linked to inflammation, we have recently developed a new Gd-based contrast agent which specifically binds to tropoelastin (TESMA), the precursor of elastin. We have successfully demonstrated the feasibility of imaging of tropoelastin in mouse models of atherosclerosis [62] and abdominal aortic aneurysm and future studies will focus on the added value of TESMA for the detection adverse myocardial remodeling after myocardial infarction.

Finally, altered levels of matrix metalloproteinases (MMPs), particularly MMP-2 and MMP-9, which are enzymes involved on the extracellular matrix remodeling, have been associated with adverse myocardial remodeling and poor prognosis after MI [63–65]. Non-invasive imaging using radiotracers enabled the quantification and localization of MMP activation in a murine model of myocardial infarction [66]. This is one of the first approaches that enables the measurement of the biological activity of enzymes involved in the remodeling process directly and, thus, could provide important prognostic information and guide treatment decisions.

## Molecular Imaging of Inflammation After Ischemia

**Fig. 4:**
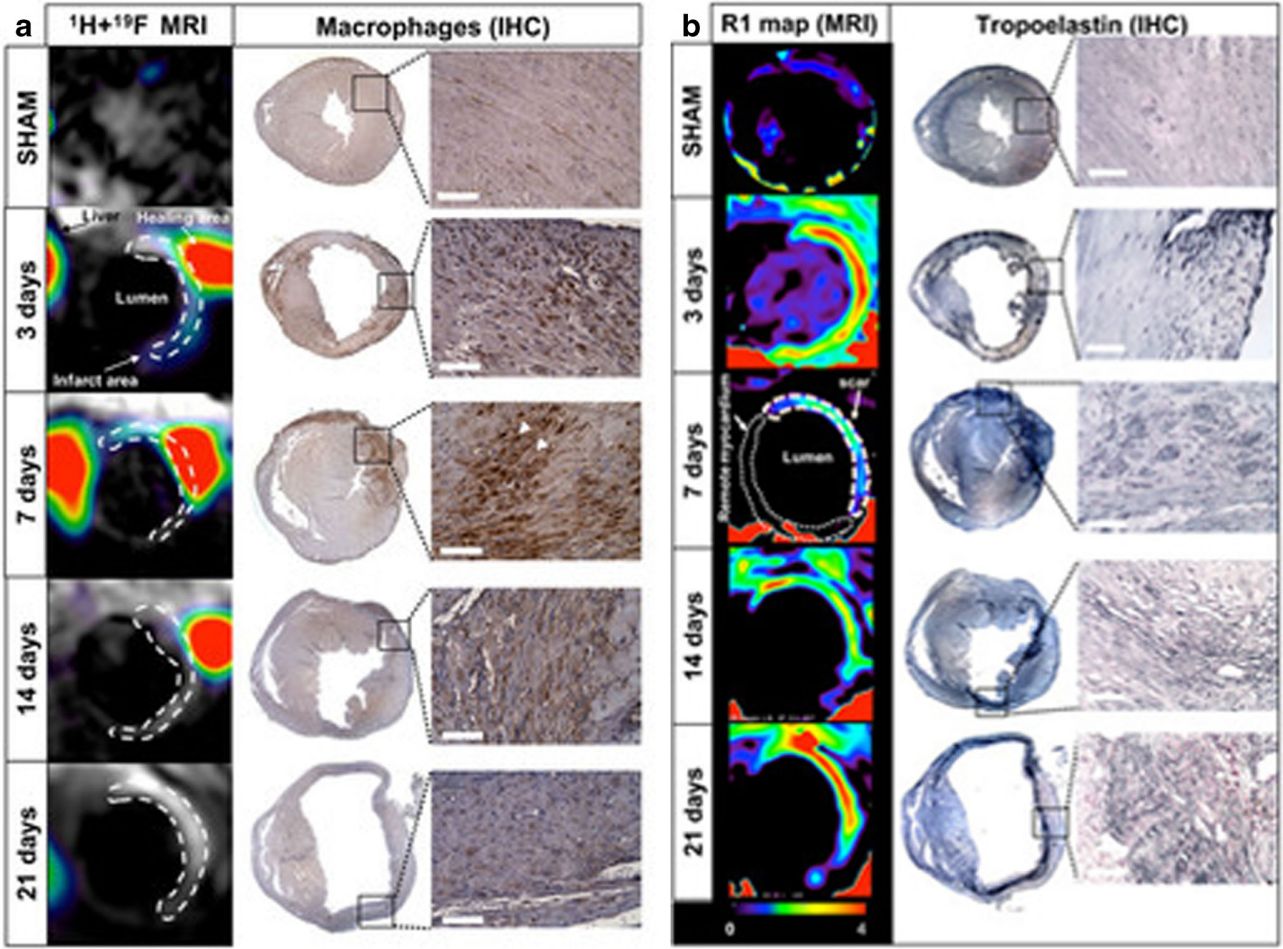
**a** Co-registered ^1^H and ^19^F short-axis images after PFC administration and macrophage immunohistochemistry (MAC-3) at different time points post-MI. **b** Representative short-axis images of relaxation rate (R_1_) maps after ESMA administration and tropoelastin immunohistochemistry of the hearts at different times points post-MI

After an ischemic event, different immune cells such as neutrophils and monocytes are released by the bone marrow and spleen and migrate into the infarcted area to trigger an inflammatory response which will contribute decisively to the healing and remodeling of the ventricle [67]. There are different ways to non-invasively image these inflammatory cells, either using their phagocytic properties (passive targeting) or by targeting specific markers expressed on the cell of interest known as active targeting. Iron oxide particles have been the choice for imaging phagocytes and successful macrophage imaging has been shown both in animal models [68–74] and humans [75–78]. The presence of macrophages at the site of injury has been best elicited via the use of superparamagnetic iron oxide nanoparticles (SPION). This method exploits the phagocytic properties of macrophages which in turn detect and internalize SPIONs as foreign bodies. SPIONs are considered unique molecular probes considering their chemical neutrality or inertness, their small size, and their excellent relaxation properties. After macrophage phagocytosis, SPIONs produce T2* shortening effects that can be detected using T2*-weighted gradient echo sequences showing hypointense areas, also referred to as signal voids, thus enabling the detection of the presence and degree of inflammation [79, 80]. An alternative way of detecting and quantifying SPION’s is by the use of susceptibility gradient mapping (SGM), which is a quantitative post processing method that measures the focal field disturbance caused by SPION’s. SGM has been successfully used to detect macrophages at different stage of atherosclerosis in mice [74]. An alternative to iron oxide particles are fluorine-based nanoemulsions (^19^F), which are also actively phagocytosed by macrophages and other phagocytic cells. An advantage of ^19^F nanoemulsions are the lack of unwanted background signal, as fluorine is not present in detectable amounts in the human body. Our group and others have demonstrated the merits of ^19^F-nanoemulsions to directly image inflammatory cells in murine models of MI [61, 81] (Fig. 4). Although this approach is very appealing due to the lack of background signal, it suffers from low signal to noise, requires dedicated dual nucleus coils, and requires high amounts of ^19^F-nanoemulsions to be injected to obtain a detectable signal. Furthermore, similar to SPION’s, the pharmacokinetics of ^19^F-nanoemulsions is complicated as they have long-retention times in blood and tissues, requiring delayed imaging (usually 24 h post injection) and thus making it more challenging to obtain approval for clinical use.

Receptor-based targeting of macrophages has also been an important area of research. Myeloperoxidase (MPO) is an inflammatory enzyme produced by neutrophils and macrophages that is highly expressed in acute MI or in culprit atherosclerotic lesions [82]. Bogdanov et al. developed a gadolinium-based contrast agent that targets MPO (MPO-Gd) [83] which was validated in murine models of ischemia and reperfusion [84]. The use of Gd-MPO allowed them to asses MPO activity in vivo in the infarcted myocardium, which is a direct measurement of the inflammatory response triggered in the heart by an acute MI. They were also able to demonstrate the beneficial effect of an anti-inflammatory therapeutical intervention, using atorvastatin, which resulted in lower levels of MPO activity [84]. Other target-specific contrast agents such as gadolinium immunomicelles targeted to the macrophage scavenger receptor CD206 [85], gadolinium-loaded LDL-based nanoparticles [86], and CX3CL1 nanoparticles [87] have been successfully tested to image and quantify macrophages in atherosclerosis. However, these agents have not been yet tested in the context of MI.

Nuclear medicine research has also been very active and focused on the development of novel radiotracers to target inflammation. ^18^F-deoxyglucose (FDG) is the best characterized and widely available tracer for imaging inflammation (metabolic activity of cells) in different diseases, such as cancer and cardiovascular diseases, among others [88]. Leukocytes that infiltrate the infarcted myocardium have very high metabolic activity and consume high concentrations of glucose. FDG imaging takes advantage of the metabolic change allowing the visualization and quantification of the inflammatory response at difference stages of the disease. FDG imaging in combination with PET/MRI technology has allowed the visualization of the biphasic nature of monocyte infiltration after an ischemic insult in a murine model of MI and in patients with acute MI [89]. In addition, FDG-PET uptake 5 days after percutaneous coronary intervention had an inverse correlation with myocardial outcome measure by MRI 6–9 months after the ischemic event in patients with cardiovascular disease [90•]. In addition, FDG signal exceeded the scar area measured by MRI, demonstrating that FDG measures the area at risk. Importantly, blood leukocyte counts correlated with both, area at risk measured by FDG-PET and scar size measured by MRI [90•]. However, cardiomyocytes have high metabolic activity and therefore produce a high FDG signal, which can mask inflammatory activity. To overcome this limitation, patients undergo special dietary requirements, including fasting or high fat meals to potentiate fatty acid metabolism and suppress cardiomyocyte glucose uptake [91]. Because this method does not always provide the desirable signal suppression from cardiomyocytes, we need new, more specific imaging radiotracers to detect and quantify inflammation. One alternative is the use of the glucose isomer mannose. Because macrophages express on their membranes the scavenger receptor known as CD206 or mannose receptor, ^18^F-deoxymannose (FDM) can be used for macrophage imaging. In addition, this receptor is highly expressed on M2-like macrophages opening a new research window not only for macrophage detection, but also for the differentiation between the two major macrophage subsets. FDM has been tested in atherosclerotic rabbits showing that the uptake obtained with FDM is not inferior to the one when using FDG [92]. However, FDM remains to be evaluated in myocardial infarction and in patients with cardiovascular disease.

**Fig. 5:**
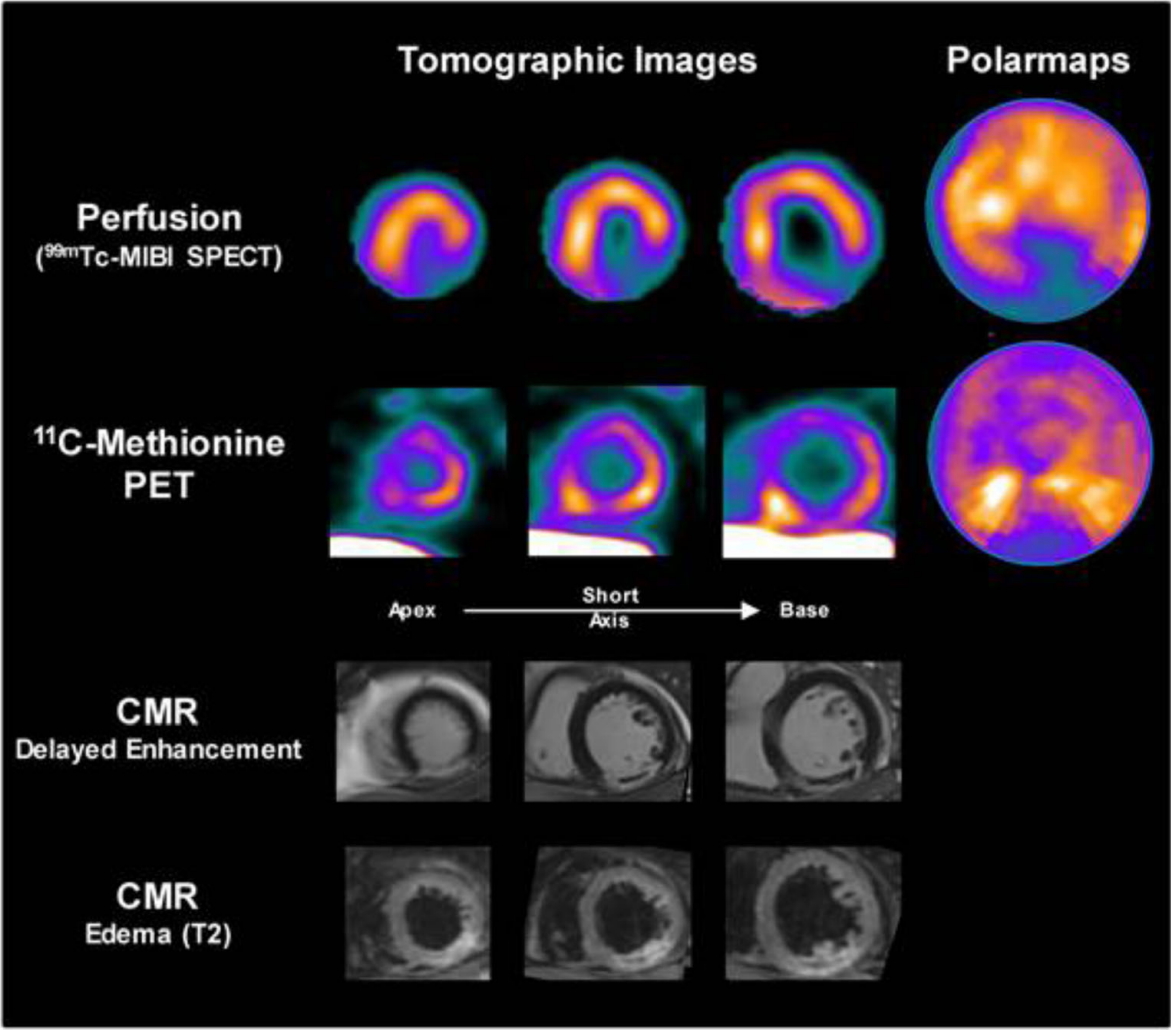
Images from a patient acquired 3 days after acute myocardial infarction and reperfusion. Perfusion SPECT and ^11^C-methionine PET cardiac images and associated polar maps display diffuse myocardial uptake with elevated ^11^C-methionine accumulation in the border zone of the perfusion defect. Cardiac magnetic resonance images confirm delayed gadolinium enhancement in the infarct region, with central no-reflow area, alongside with edema in the infarct region on T2-weighted images

^11^C-methionine is a well-characterized PET radiotracer commonly used for detection of brain tumors. Thackeray et al. demonstrated in a murine model of MI that PET imaging with ^11^C-methionine allows for detection of inflammation in the injured myocardium at the early stages post-MI and observed a signal decline over 7 days which was paralleled by a decrease of activated inflammatory cells [93] (Fig. 5). In a clinical proof-of-concept study, ^11^C-methionine uptake was observed in patients with acute MI up to 2 weeks after reperfusion [94].

The somatostatin receptor type 2 (SSTR2) is also highly expressed in activated macrophages, especially in M1-like macrophages, and is routinely used for imaging neuroendocrine tumors. Two imaging agents that are a newer generation of somatostatin analogs have been developed, known as ^68^Ga-DOTATATE and ^68^Ga-DOTATOC. ^68^Ga-DOTATATE has higher affinity to the SSTR2, while ^68^Ga-DOTATOC has higher affinity to SST5. Both radiotracers have shown different imaging benefits compared to FDG, as it not only presents superior accuracy and sensitivity, but also allows characterization of whole-body SSTR expression [95]. ^68^Ga-DOTATATE has shown very promising results for the detection of inflammation in atherosclerosis [96]; however, due to the very quick metabolism and blood clearance, no conclusive results were observed in a murine model of MI [97]. In contrast, with ^68^Ga-DOTATOC, the elevation of SSTR2 in the infarcted myocardium at 3 and 10 days post-acute MI has been observed in patients [98].

C-X-C chemokine receptor 4 (CXCR4) is a receptor involved in leukocyte migration and recruitment to injured tissue, such as the infarcted myocardium. ^68^Ga-pentixafor is a PET radiotracer that has shown promising results for the detection of inflammation in a murine model of MI. Tracer uptake in the infarcted myocardium was proportional to leukocyte infiltration as detected by flow cytometry [99]. In the same study of 12 patients with myocardial infarction, this radiotracer showed heterogeneous uptake at time points between 2 and 8 days post-MI, suggesting a patient specific modulation of the chemokine response [99, 100]. Studies with larger number of patients are now required to better understand the chemokine response provided by the CXCR4 receptor after MI.

Although several strategies have been developed to image macrophages, none of them has had the ability to clearly distinguish between macrophage subsets. Thus, the development of M1 or M2 specific probes remains an interesting challenge on the horizon.

## Conclusion

Important advances in imaging the molecular processes associated with the ischemia-induced myocardial injury and resulting remodeling have been made in the last decade. The development of new target-specific contrast agents, with some of them currently being clinically validated, could open a new era in the management of patients with myocardial infarction, thereby allowing better patient stratification and enable more personalized treatments. However, intense research is still needed at both the preclinical and clinical level to develop not only more specific contrast agents, but also to better understand the biological information provided by those reagents. For clinical translation, further validation of these new agents needs to be performed in larger patient cohorts to evaluate the diagnostic and prognostic value. The discovery and development of new agents able to differentiate between the different subsets of leukocytes, monocytes, and macrophages present at different time points in the infarcted myocardium could open new avenues for more personalized treatments, prognosis and outcome studies.
